# pH and NaCl Optimisation to Improve the Stability of Gold and Silver Nanoparticles’ Anti-Zearalenone Antibody Conjugates for Immunochromatographic Assay

**DOI:** 10.3390/mps6050093

**Published:** 2023-10-03

**Authors:** Thasmin Shahjahan, Bilal Javed, Vinayak Sharma, Furong Tian

**Affiliations:** 1 School of Food Science and Environmental Health, College of Sciences and Health, Technological University Dublin, D07 H6K8 Dublin, Irelandfurong.tian@tudublin.ie (F.T.); 2 Nano Lab, FOCAS Research Institute, Technological University Dublin, D08 CKP1 Dublin, Ireland; 3 RELX Elsevier, D18 X6N2 Dublin, Ireland

**Keywords:** antibody conjugates, biosensing, bioreporter, lateral flow devices, nanoparticles aggregation, pH

## Abstract

The aim of this research is to define optimal conditions to improve the stability of gold and silver nanoparticles’ anti-zearalenone antibody conjugates for their utilisation in lateral flow immunochromatographic assay (LFIA). The Turkevich–Frens method was used to synthesise gold nanoparticles (AuNPs), which were between 10 and 110 nm in diameter. Silver nanoparticles (AgNPs) with a size distribution of 2.5 to 100 nm were synthesised using sodium borohydride as a reducing agent. The onset of AuNP and AgNP aggregation occurred at 150 mM and 80 mM NaCl concentrations, respectively. Stable Au and Ag nanoparticle–antibody conjugates were achieved at 1.2 mM of K_2_CO_3_ concentration, which corresponds to the pH value of ≈7. Lastly, the highest degree of conjugation between Au and Ag nanoparticles and anti-zearalenone antibodies was at 4 and 6 µg/mL of antibody concentrations. The optimisation of the conjugation conditions can contribute to better stability of nanoparticles and their antibody conjugate and can improve the reproducibility of results of bioreporter molecules in biosensing lateral flow devices.

## 1. Introduction

The detection of zearalenone via lateral flow immunochromatographic assays plays a significant role in food safety and agriculture. Zearalenone is a mycotoxin, which is produced by certain fungal species and poses significant risks to both human and animal health when present in food and feed beyond a certain level. Rapid detection of zearalenone through LFIA provides a cost-effective, user-friendly, and on-site screening device for farmers, food processors, and regulatory authorities. It enables the timely identification of contaminated crops and products, which facilitates immediate interventions to mitigate health risks and economic losses. Moreover, the portability and simplicity of the assay make it an essential tool in monitoring and ensuring the safety of our food supply chain. 

In the LFIA, the signal labels are generally conjugated with a detection agent, such as antibodies for the specific binding to the target analyte, like mycotoxins. This allows a visible signal to be generated by the recognition elements to indicate the presence of the antigen. The challenge in immobilising antibodies onto AuNPs or AgNOs is avoiding aggregation and ensuring that the antibodies are orientated correctly to maintain the functionality and the accessibility of their paratopes to conjugate with the antigens or analyte [1]. In order for the signal labels to precisely and accurately detect zearalenone, they must fulfil a range of criteria, which include having high stability, exhibiting little or no non-specific binding, being cost-effective, and forming reproducible and efficient conjugates without compromising the functionality and activity of the detection molecule [2].

To effectively use antibody–NP (Ab-NP) bioconjugates for biosensing, it is vital to develop robust and reliable techniques to ensure that the produced biosensor is reproducible, selective, and sensitive. An efficient bioconjugation approach must preserve the colloidal stability of the nanoparticles (NPs) while maintaining the capacity of Ab-NP bioconjugates to identify their target antigen [3]. Nanoparticles can be conjugated by physical adsorption, which is typically the preferred method for LFIA applications. It involves immobilising detection molecules onto noble metal nanoparticle’s surfaces via hydrophobic, electrostatic interactions, hydrogen bonds, and Van der Waals forces [4]. The optimisation of this process can be achieved by testing different pH values near the isoelectric point of the binding molecule [5]. However, physical adsorption can lead to unreliable results due to the erroneous orientation of detection molecules, leading to the blocking of binding sites and poor reproducibility [6]. Controlling the pH between 7.5 and 8.5 can help control the orientation of antibodies and improve direct binding to citrate-stabilized NPs [7]. 

To determine the optimal quantity of antibody for adsorption, the flocculation test is conducted. This test identifies the minimum amount of antibodies needed to maintain the stability of NPs against salt-induced aggregation. Usually, 10% of NaCl is used for the flocculation test, which is followed by measuring the colour change in the colloidal solution or the absorbance of the solution at its λ max [8]. An optimal conjugate can be achieved by adding varying concentrations of antibodies to the AuNPs/AgNPs. If the conjugate aggregates upon contact with sodium chloride, it indicates that the mixture does not contain a sufficient number of conjugated antibodies, resulting in a colour change in the solution from red to blue [9,10].

Changes in the environment of AuNPs/AgNPs often result in the formation of aggregates. The term aggregate is used to refer to individual nanoparticles that have interacted with each other to form a larger super-structure without altering the shape or size of individual nanoparticles [11]. As maintaining the stability of conjugates in LFIA strips is crucial, it is essential to gain a deeper understanding of the nanoparticle conjugates for the effective optimisation of their performance in lateral flow assay. This can be achieved by characterising standard nanoparticles and conjugates using analytical techniques and measuring different parameters such as size, shape, zeta potential, absorbance, and optical density to monitor their stability [12].

The size of nanoparticles plays a crucial role in the sensitivity of LFIA. If aggregation occurs, the colour of gold nanospheres in suspension changes from wine-red to darker shades, affecting the intensity of the lines on the strip [13]. According to the study conducted by Sahoo and Singh [14], the size of nanoparticles can be controlled by adjusting parameters such as the concentration of sodium citrate, pH, and temperature. Nanospheres with diameters in the range of 20–40 nm are commonly used in optimising parameters for LFIA sensitivity, as larger nanoparticles can provide enhanced colour observation; however, they are less stable [14]. As aggregation is a challenge during conjugation, monitoring the size of standard nanoparticles as well as conjugates can aid in determining their aggregation state [15]. 

The aim of this project was to develop stable gold and silver nanoparticle anti-zearalenone antibody conjugates. It involved the synthesis and characterisation of AuNPs and AgNPs and their anti-zearalenone antibody conjugates. Three main studies were performed for optimising nanoparticle–antibody conjugate conditions. Various physical and optical parameters were measured using DLS, SEM, and UV-visible spectroscopy. These include absorbance, optical density, particle shape, particle size, polydispersity index (PDI), and zeta potential. In the first study, the nanoparticles were studied under various concentrations of NaCl in a 96-well plate to examine the aggregation behaviour of nanoparticles in an alkaline environment. A second study was performed to determine the optimum pH at which successful nanoparticle–antibody conjugation can be achieved. It involved the conjugation of nanoparticles with 2 µg/mL of antibodies under varying concentrations of K_2_CO_3_ to adjust the pH of the reaction mixture. The NaCl concentration at which the onset of nanoparticle aggregation occurred was added to determine the success of the conjugation process. The stability of the conjugates and the aggregation state were evaluated via the characterisation of conjugates. A third study was carried out where the concentration of K_2_CO_3_ and NaCl were kept constant, and the antibody concentration was varied to determine the concentration at which maximum nanoparticle–antibody conjugation can be achieved under a controlled environment. Therefore, this study provides a novel comparative systematic analysis to develop stable gold and silver nanoparticle–antibody conjugates to improve overall performance and to enhance the accuracy of LFIA test results.

## 2. Material and Methods

### 2.1. Materials

Gold (III) chloride trihydrate (HAuCl_4_⋅3H_2_O), trisodium citrate, AgNO_3_, NaBH_4,_ NaCl, K_2_CO_3,_ and sodium hydroxide (NaOH) were purchased from Sigma-Aldrich, Dublin, Ireland. Anti-zearalenone [11C9] mouse monoclonal antibodies were procured from Abcam. Deionised water (DI) was produced by using an Elix Reference Water Purification System from Millipore, Ireland, and was used for the preparation of the solution. All the chemicals were used as received from the supplier without further purification or modification.

### 2.2. Gold Nanoparticles Synthesis

According to the procedure outlined by Turkevich et al. (1951), colloidal AuNPs were produced using the Turkevich–Frens technique [16]. A 250 mL glass beaker was filled with 250 µL of 50.5 mM HAuCl_4_ stock solution and 94.75 mL of deionised water. The mixture was heated to a temperature of 100 °C and then placed on a hot stirrer. At a moderate rate of mechanical stirring, 5 mL of 1% trisodium citrate was added to the boiling solution at a rate of 1 mL/s. The solution started out faintly pink after two minutes and steadily became darker over the course of approximately eight minutes. After a deep wine-red colour appeared, the reaction was stopped by removing it from the water bath and allowed to cool down at room temperature.

### 2.3. Silver Nanoparticles Synthesis

AgNPs were generated using the procedure outlined in [17]. An amount of 30 mL of deionised water was used to dissolve 0.0023 g of NaBH_4_ to create a final concentration of 0.002 M. AgNO_3_ was dissolved in 10 mL of deionised water to a final concentration of 0.001 M using 0.0017 g of the reagent. The 0.002 M NaBH_4_ solution was incubated for 20 min in an ice batch. The NaBH_4_ solution was subsequently mixed with 0.001 M of AgNO_3_ at a rate of 1 mL/s. A cold magnetic stirrer was used to vigorously stir the reaction mixture. The solution turned pale yellow following the addition of 2 mL of AgNO_3_, and it eventually turned bright yellow after incorporating the entire quantity of AgNO_3_. 

### 2.4. Gold and Silver Nanoparticles Aggregation Study under the Influence of Salt

A total of 150 µL of colloidal gold/silver nanoparticles were loaded in a 96-well plate. An amount of 150 µL of deionized water was added to the first three wells as the control. Then, duplicate additions of 150 µL of NaCl at various concentrations (20, 40, 60, 80, 100, 180, 200, and 400 mM) were made. For approximately two minutes, the plate was left to incubate at room temperature. The colour changed after two minutes. The SpectroMax plate reader was used to quantify the OD and absorbance [12].

### 2.5. Investigating pH Changes by Using Various K_2_CO_3_ Concentrations against Silver and Gold Nanoparticles

A micropipette was used to add 150 µL of colloidal gold/silver nanoparticles into the wells of a 96-well plate. An amount of 150 µL of deionized water was placed into the first three wells of the first row for control 1. Then, for control 2, 150 µL of deionised water and 2 µg/mL of antibody solution were pipetted into the first three wells of the second row. To achieve the following concentrations—0.0 mM, 0.2 mM, 0.4 mM, 0.6 mM, 0.8 mM, 1.0 mM, and 1.2 mM—and a final volume of 100 µL, various amounts of a 10 mM K_2_CO_3_ stock solution were then added to subsequent rows. To each well, 2 µg/mL of the antibody solution was added. To ensure the antibodies were evenly distributed throughout the nanoparticle solution, the mixtures were reverse pipetted. For approximately 20 to 25 min, the plate was set aside at room temperature. Next, to the Au and AgNPs, 200 mM and 80 mM NaCl, respectively, were added. The OD and absorbance were measured using the SpectroMax plate reader once colour change occurred [12].

### 2.6. Study of Silver and Gold Nanoparticles at Different Antibody Concentrations

A micropipette was used to add 150 µL of colloidal gold/silver nanoparticles into the wells of a 96-well plate. An amount of 150 µL of deionized water was added into the first three wells of the first row for control 1. Then, for control 2, 150 µL of deionized water and 2 ug/mL of antibody solution were pipetted into the first three wells of the second row. With the exception of the control wells, 100 µL of K_2_CO_3_ optimal concentration was introduced to each well. The antibody solution was then added in different concentrations. Amounts of 2 and 4 µg/mL were employed for AuNPs. For AgNPs, antibodies at concentrations of 2, 4, and 6 µg/mL were used. To ensure that the antibodies were evenly distributed throughout the nanoparticle solution, the mixtures were reverse pipetted. For approximately 20 to 25 min, the plate was left to rest at room temperature. Then, for the addition of Au and Ag NPs, 200 mM and 80 mM NaCl were added, respectively. The OD and absorbance were measured using the SpectroMax plate reader once colour change occurred.

### 2.7. Particle Characterisation

On a Malvern Instrument Nano-Zetasizer, DLS measurements were performed. In order to prepare the samples, 900 μL of deionised (DI) water and 100 μL of the nanoparticle colloidal sample from Section 2.2 or Section 2.3 were mixed in a disposable plastic cuvette. Prior to the measurement, the samples were allowed to adjust to 25 °C for approximately 2 min. After three successive runs with each sample, graphs of the size distribution by intensity were analysed. 

A pipette was utilised to transfer 800 µL of the sample from the disposable cuvette to a Malvern Panalytical Folded Capillary Zeta Cell for ZP measurement. The UV-visible spectrophotometer did not require dilution to measure absorbance. One millilitre of the sample was added to a small cuvette before being placed in the sample container. Transmission Electron Microscopy (TEM) was used to investigate the morphology and size of AuNPs and AgNPs [9,18,19,20].

## 3. Result and Discussion

### 3.1. Characterisation of Gold and Silver Nanoparticles

**Figure 1:**
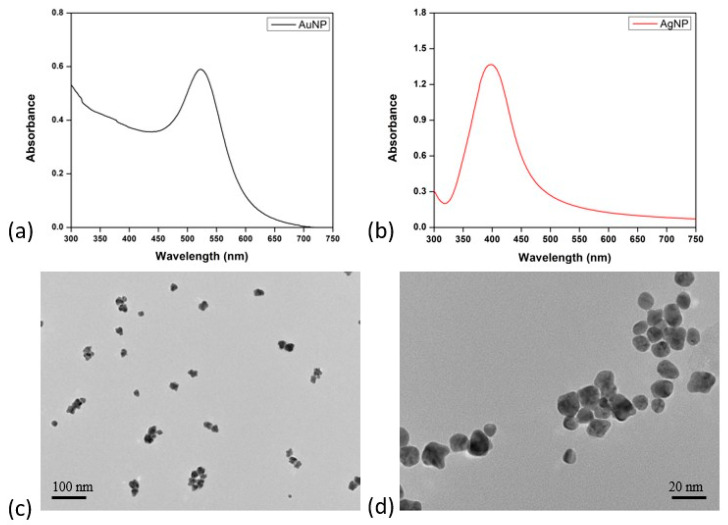
Characterisation of gold and silver nanoparticles. (**a**) The UV-visible spectra of AuNPs, (**b**) the UV-visible spectra of AgNPs, (**c**) a TEM image of AuNPs, and (**d**) a TEM image of AgNPs.

The synthesis of AuNPs and AgNPs was achieved using trisodium citrate as the reducing agent for AuNPs and silver nitrate for AgNPs. The reduction reactions were conducted under carefully optimised conditions including controlled temperature and reaction time to facilitate the nucleation and growth of stable nanoparticles. The particle spectra and images are presented in Figure 1.

Figure 1a,b show the UV-visible spectra of the synthesised silver and gold NPs exhibit distinct absorption peaks in the 300 nm to 800 nm spectral range. Evident within these spectra are well-defined characteristic plasmon resonance peaks observed at 520 nm for AuNPs and 400 nm for AgNPs. These plasmon resonance peaks indicate the collective oscillation of electrons on the nanoparticle surface. This confirms the successful formation of colloidal Ag and Au nanoparticles.

The TEM images revealed that both nanoparticles display a spherical morphology with a relatively narrow size distribution (Figure 1c,d). AgNPs exhibited diameters ranging from 17.6 nm to 25.8 nm, while AuNPs demonstrated an average diameter spanning from 5.95 nm to 11.9 nm. When conjugating the AuNPs and AgNPs with antibodies, the spherical morphology of the synthesised AuNPs and AgNPs may be advantageous. This is primarily attributed to the potential enhancement in achieving a well-orientated distribution of antibodies across the nanoparticle surface. Consequently, this may potentially enhance both the binding efficiency and sensitivity [21].

The size of nanoparticles holds the potential to significantly influence the overall performance of the application in lateral flow assay [22]. Dynamic Light Scattering (DLS) was employed to determine the hydrodynamic size distributions of the nanoparticles. The obtained size distribution by intensity profiles, which indicates that the AuNPs exhibited a size range of 9 nm to 120 nm, while the AgNPs were observed to range from 2.5 nm to 120 nm. These findings denote the presence of a well-dispersed population. The range suggests that the nanoparticles are predominantly polydisperse. AgNPs have a relatively broader range of hydrodynamic sizes compared to AuNPs. The introduction of larger nanoparticles could be advantageous. According to their findings, increasing the nanoparticle size to as high as 115 nm resulted in a significant drop in the limit of detection (LOD) [23]. The polydispersity index (PDI) and Zeta Potential (ZP) values are shown in Table 1. 

Au and Ag nanoparticles had zeta potentials of −26.3 ± 4.6 mV and −20.07 ± 0.5 mV, respectively, showing strong negative surface charges. They are relatively stable as zeta potential values other than −30 mV to +30 mV are deemed stable [24]. Furthermore, the zeta potential of the AuNPs was comparatively stronger than that of the AgNPs, indicating better stability. 

It was reported that PDI greater than 0.7 is an indication of aggregated nanoparticles in the solution [25]. The current result shows a PDI value of 0.2 for the AuNPs, indicating a narrow size distribution, meaning that the majority of the particles are similar in size. On the other hand, a PDI value of 0.5 was obtained for the AgNPs, which is typically acceptable and suggests a broader size distribution.

### 3.2. Analysis of Gold and Silver Nanoparticles Aggregation Behaviour in Differing Alkaline Environments

**Figure 2:**
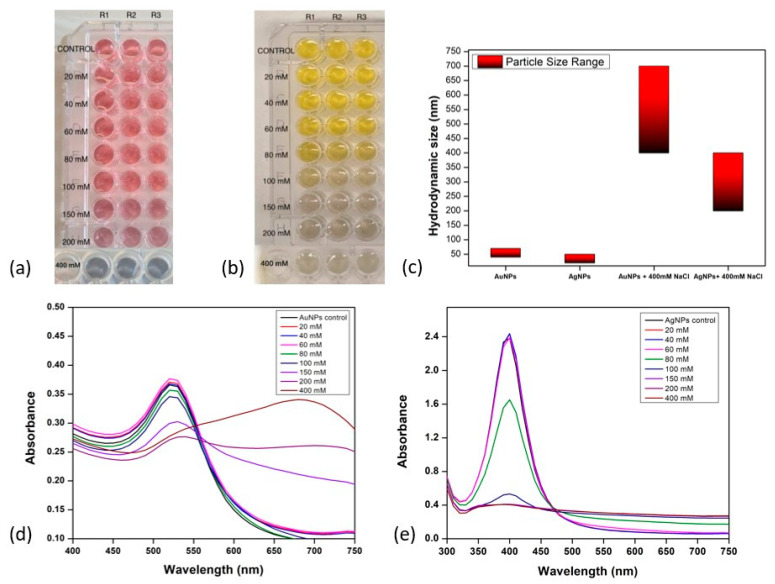
Gold and silver nanoparticles aggregation behaviour in differing alkaline environments. (**a**) Image of AuNP with series of NaCl solution in the 96 well plate. (**b**) Image of AgNP with series of NaCl solution in the 96 well plate. (**c**) Histogram of the hydrodynamic size of AuNPs and AgNPs with and without 400 mM NaCl. (**d**) The spectra of AuNPs under different concentrations of NaCl. (**e**) The spectra of AgNPs under different concentrations of NaCl.

Prior to antibody conjugation, the stability of the synthesised Au and Ag nanoparticles was investigated in NaCl concentrations ranging from 20 mM to 400 mM. The aim was to investigate the effects of different NaCl levels on nanoparticle stability. For accuracy, the experiment used a 96-well plate format with triplicate samples (Figure 2). 

Figure 2 depicts the aggregation behaviour of AuNPs (Figure 2a,d) across various NaCl concentrations. At NaCl concentrations of 20 mM, 40 mM, 60 mM, 80 mM, and 100 mM, AuNPs displayed no aggregation while retaining their typical brilliant red colour and stability. However, around 150 mM, a shift from red to pink-purple occurred, suggesting that the physical and chemical characteristics had changed in response to unfavourable alkaline conditions. At a NaCl concentration of 150 mM, the stability of AuNPs was disturbed, resulting in aggregation. The observed grey colour indicates that saturation was achieved at 400 mM. Similar trends were observed for AgNPs, with slight variations. The onset aggregation of AgNPs was observed at 80 mM of NaCl, characterised by a transition from brilliant yellow to light grey colouration (Figure 2b,e). Following that, saturation at 150 mM of NaCl was observed, resulting in the development of dark grey nanoparticles. These varied observations together highlight the complex relationship between nanoparticle stability and NaCl concentration.

**Figure 3:**
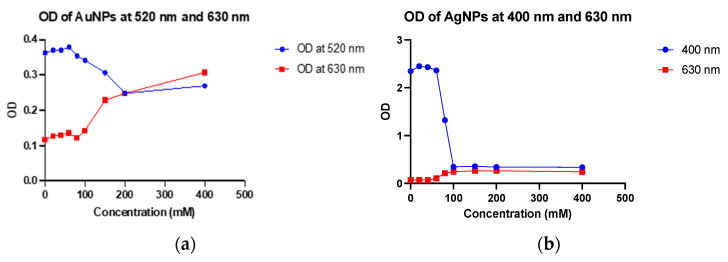
Optical density (OD) of AuNP and AgNP at varying NaCl Concentrations. (**a**) OD of AuNP was taken at 520 nm and 630 nm. OD decreases at 520 nm and increases at 630 nm; (**b**) OD of AgNP at varying NaCl Concentrations. OD decreases at 400 nm and increases slightly at 630 nm. Note: 0 mM (control), 20. NaCl Concentrations of 0 mM (control), 20 mM, 40 mM, 60 mM, 80 mM, 100 mM, 150 mM, and 400 mM were used.

For additional insight into the behaviour of the AuNPs in the alkaline environment, the optical density (OD) was measured at 520 nm and 630 nm to monitor changes in their SPR properties as a function of NaCl concentration. The OD measurements obtained at 520 nm and 630 nm were plotted on a graph, which is shown in Figure 3.

The OD at 520 nm decreased slightly at NaCl concentrations 20 mM to 100 mM, while at NaCl concentrations 150 mM to 400 mM, a more significant decrease was observed (Figure 3a). This indicated a decrease in the concentration of dispersed nanoparticles as the NaCl concentration increased due to increased aggregation. The SPR of the nanoparticles slightly shifted to a longer wavelength at NaCl concentrations of 20 to 100 mM. These shifts were more significant as the NaCl concentration increased beyond 100 mM; hence, a significant decrease in OD was observed. This is consistent with the red shift in the SPR peak observed in Figure 2d. These shifts in SPR occurred as a result of the interaction of Na+ and Cl- ions with the surface charge of nanoparticles, with stronger interactions occurring at higher NaCl concentrations. Conversely, at 630 nm, the OD increased with increasing NaCl concentration (Figure 3b). This was clearly due to the shift in the SPR of nanoparticles to longer wavelengths. The increase in OD became more significant at 150 mM, which is the concentration at which the onset of nanoparticle aggregation occurs, as determined by the corresponding SPR peak on the UV-visible absorption spectra.

The hydrodynamic sizes of both gold and silver nanoparticles (without NaCl influence) and with NaCl were measured using dynamic light scattering analysis (Figure 2c). The data reveals the size of AuNPs in the control sample was in the range of 40–70 nm and of AgNPs was 20–40 nm. After the addition of NaCl at the concentration of 400 mM, a change in colour was observed, which shows the aggregation of both AuNPs and AgNPs. The aggregation was confirmed by checking the hydrodynamic size, zeta potential, and PDI values of dispersions of AuNPs and AgNPs. The size was found to be in the range of 450–700 nm for AuNPs and 200–400 nm in the case of AgNPs. PDI values were also found to be close to 1, along with zeta potential close to zero, indicating unstable and aggregated dispersions. The colour changed to grey, indicating that high NaCl concentration led to aggregation. Hence, for further experiments, the concentration of NaCl used was 150 mM for AuNPs and 80 mM for AgNPs.

### 3.3. Influence of Colloidal Solution pH on Antibody Conjugation to Gold and Silver Nanoparticles

**Figure 4:**
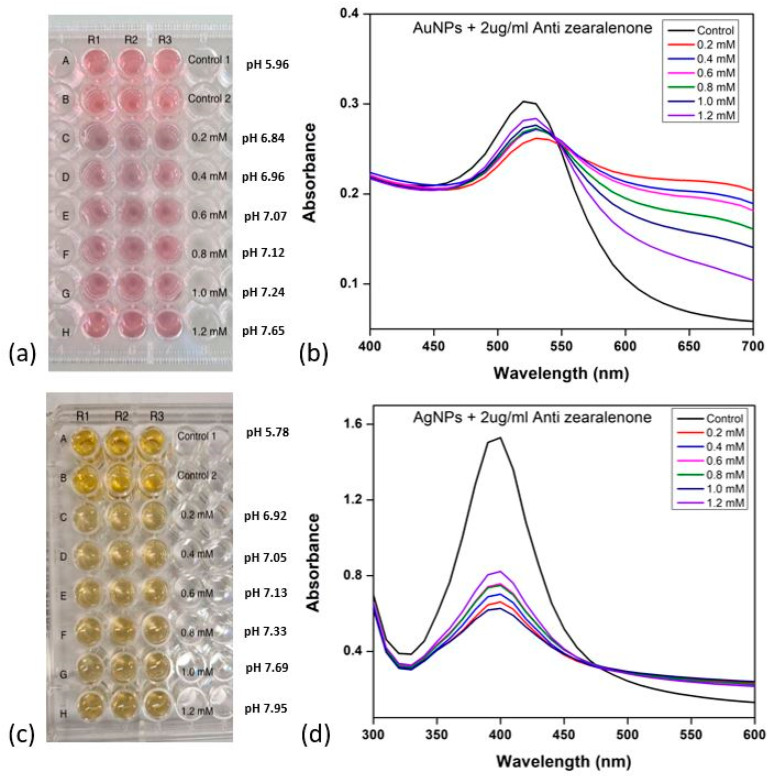
Gold and silver nanoparticle aggregation behaviour in differing alkaline environments. (**a**) Image of AuNP with the series anti-zearalenone solution in the 96-well plate. (**b**) The spectra of AuNP at varied anti-zearalenone concentrations. (**c**) Image of AgNP with the series anti-zearalenone solution in the 96-well plate. (**d**) The spectra of AgNP at varied anti-zearalenone concentrations.

The effect of varying pH levels on the interaction between the nanoparticles and antibodies was investigated. K_2_CO_3_ was employed to adjust the pH, with concentrations ranging from 0.2 mM to 1.2 mM. To verify the conjugation, a suboptimal NaCl concentration of 150 mM was introduced for AuNPs and 80 mM for AgNPs. The study aimed to identify the optimal pH for antibody–nanoparticle (Ab-NP) conjugation. The pH of colloidal Au and Ag nanoparticle solutions was regulated using K_2_CO_3_, while antibody concentration remained constant at 2 µg/mL. The details of the K_2_CO_3_ concentrations used to adjust the pH of the colloidal AuNPs and AgNPs solutions are provided in Figure 4a,c, respectively. Figure 4 shows the effect of varying pH levels maintained via K_2_CO_3_ on the interaction between the nanoparticles and antibodies. Figure 4a,c illustrates visually monitored solutions for colour changes in the 96-well plate.

The image in Figure 4a shows the plate used for the colourimetric assay of Ab-AuNP conjugates. Colour changes occurred at concentrations of 0.2, 0.4, 0.6, 0.8, and 1 mM, signifying nanoparticle aggregation after NaCl addition. This indicated unsuccessful antibody–AuNP conjugation due to suboptimal pH. Lower K_2_CO_3_ concentrations created a low-pH environment, causing electrostatic bridging between antibodies and negatively charged citrate-capped nanoparticles, resulting in aggregation. At 1.2 mM K_2_CO_3_, corresponding to the pH 7.65, the nanoparticles exhibited minimal colour change compared to the control, indicating successful antibody coating onto nanoparticle surfaces.

To investigate the effect of pH on AuNP optical properties, nanoparticle absorbance was measured using UV-visible spectroscopy (300–750 nm). Figure 4b depicts the obtained absorbance spectra. SPR peaks at around 520 nm, similar to standard AuNPs, were identified. Control 1 (AuNPs and deionised water) and Control 2 (AuNPs, deionised water, and antibodies) exhibited no changes in SPR, optimal antibody orientation on the nanoparticle surface, and stability. As the reaction pH decreased, slight redshifts appeared, suggesting augmented nano-moiety size. Changes in interparticle distances and dielectric constants of the surrounding medium are two causing factors. Lower pH resulted in larger, less defined SPR peaks, indicating destabilisation and aggregation of nanoparticle conjugates. The SPR peak closely mirrored the control peaks at 1.2 mM K_2_CO_3_, indicating stable antibody–AuNP conjugation in solution.

Figure 4c is an image of the 96-well plate employed for examining AgNPs. Control 1 contains AgNPs and deionized water, and Control 2 also includes antibodies. AgNPs followed a similar aggregation pattern to AuNPs, aggregating at 0.2, 0.4, 0.6, 0.8, and 1 mM, evident by colour shifts from bright yellow to pale yellow to light grey. Retention of bright yellow colour at 1.2 mM, which corresponds to pH 7.95, suggested optimal K_2_CO_3_ concentration for AgNPs-Ab conjugation.

The particle size distribution for control 1 (AuNPs and deionised water) was reported between 10 to 140 nm, while the values changed from 8 to 90 nm for control 2 (AuNPs, deionised water, and antibodies). At 1.2 mM K_2_CO_3_, the particle size distribution increased from 10 to 410 nm following conjugation, which shows an increase in the nanoparticle standard size and indicates the effective coverage of the nanoparticle’s surface with the antibodies. Figure 4d presents UV-visible absorbance spectra of Ab-AgNP complexes at varied K_2_CO_3_ concentrations. The SPR peaks are positioned around 400 nm. However, a significant change in peak shape and intensity is observed at low concentrations of K_2_CO_3_, indicating aggregation stability. Conjugates showed a significant absorbance drop at 400 nm for K_2_CO_3_ concentrations 0.2 mM to 1.2 mM, with 1.2 mM exhibiting a slightly smaller yet well-defined peak, suggesting stability. Table 2 illustrates the zeta potential (ZP) and PDI of AuNPs in control 1, control 2, and at 1.2 mM of K_2_CO_3_ concentration.

Control 1, control 2, and Ab-AuNP at 1.2 mM K_2_CO_3_ all had significantly negative zeta potential values of −33.4 ± 8.2 mV, −30.9 ± 10.6 mV, and −23.2 ± 1.5 mV, indicating highly stable conjugates in Table 2. A slight reduction in ZP at 1.2 mM of K_2_CO_3_ suggested a modest decrease in surface charge, presumably due to pH-driven changes in ionic strength influencing electrostatic interactions between nanoparticles in the surrounding medium. The Polydispersity Index (PDI) values were 0.229, 0.329, and 0.347 (Table 2). 

Notably, the conjugates at optimum pH had a larger PDI, indicating a greater variance in nanoparticle size and enhanced solution heterogeneity [26,27]. This corresponds to the increased hydrodynamic diameter range of single AuNP–antibody conjugates. Similar results were obtained for the AgNPs and their antibody conjugates at the 1.2 mM concentration of K_2_CO_3_ (Table 3).

**Figure 5:**
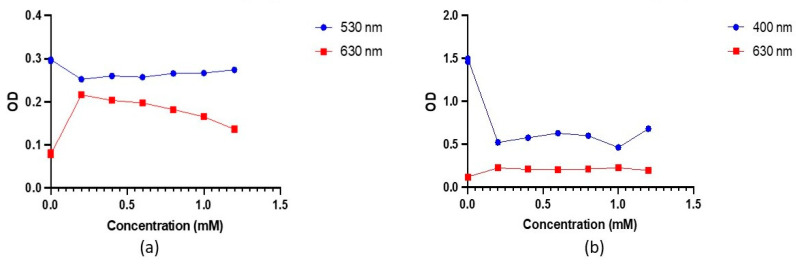
Optical density (OD) of AuNPs and AgNPs at varying K_2_CO_3_ concentrations. (**a**) OD of AuNP was taken at 530 nm and 630 nm. OD decreases at 520 nm and increases at 630 nm. (**b**) Optical density (OD) of AgNP at K_2_CO_3_ concentrations. Note: 0 mM (control at K_2_CO_3_ concentrations 0.2 mM, 0.4 mM, 0.6 mM, 0.8 mM, 1.0 mM, and 1.2 mM.

To gain further insights into antibody and nanoparticle behaviour across varying pH, OD measurements were taken at 530 nm and 630 nm on different concentrations of K_2_CO_3_ (Figure 5).

At 530 nm, a decrease in OD values was observed, with a notable decrease from control 1 to control 2. As pH increased, OD increased consistently. This pattern is mirrored in the OD measurements at 630 nm—a significant increase from control 1 to control 2—followed by a steady decrease with rising pH. The variations in pH influence the surface charge of the nanoparticles, impacting the adsorption of antibodies. These changes in surface properties can impact the SPR phenomenon, causing shifts towards longer wavelengths as a result of more prevalent repulsive interactions between particles. This is consistent with the redshift in SPR peaks at low K_2_CO_3_ concentrations observed in the UV-visible spectra. The behaviour observed at both wavelengths suggests that antibody–nanoparticle interactions are closely connected to pH-induced changes in surface charge and nanoparticle stability (Figure 5a).

OD measurements of AgNPs-Ab conjugates in Figure 5b reveal a decrease at lower pH and an increase at 1.2 mM at 400 nm. Contrastingly, variations at 630 nm are minimal. For conjugates at 1.2 mM K_2_CO_3_ concentration, the DLS data demonstrate a broad size distribution of 10 to 400 nm, highly negative ZP of −33.6 ± 1.11 mV, and slightly higher PDI of 0.312 compared to the controls (Table 3). These findings are similar to that of AuNPs antibody conjugates in this study, providing evidence of successful antibody binding for stable conjugates at 1.2 mM of K_2_CO_3_ concentration. 

Kasoju et al. [12] found that the onset of nanoparticle aggregation occurred at 40 mM and reached saturation at 80 mM. This differs from the findings of this study, as the nanoparticles began aggregating at 80 mM and reached saturation at 400 mM. This means that the nanoparticles in this study have a higher stability, are more resistant to aggregation, and require higher NaCl concentrations to induce aggregation compared to the AuNPs synthesised by Kasoju et al. [12]. Additionally, the higher concentration range for saturation suggests that AuNPs have a comparatively higher sensitivity to changes in NaCl concentration. In this study, the AuNPs size ranged between 9 and 120 nm, while the AuNPs used by Kasoju et al. [12] were approximately 18 to 20 nm. The difference in aggregation behaviour can be attributed to the size distribution of the particles, as particle size plays an important role in their stability and sensitivity to aggregation [28]. As the nanoparticles in this study were larger, they exhibited higher stability and began aggregating at a relatively higher NaCl concentration. This is advantageous for the use of these conjugates as they would be less likely to aggregate during migration on the sample pad, therefore ensuring better dispersion and enhanced signal generation for the lateral flow test strips. However, it is important to note that if the size of NPs is too large and excessively high NaCl concentrations are required to reach saturation, it could result in reduced assay sensitivity [29,30].

### 3.4. Stability Study of Gold and Silver Nanoparticles at Varying Antibody Concentrations under Controlled pH and NaCl Conditions

**Figure 6:**
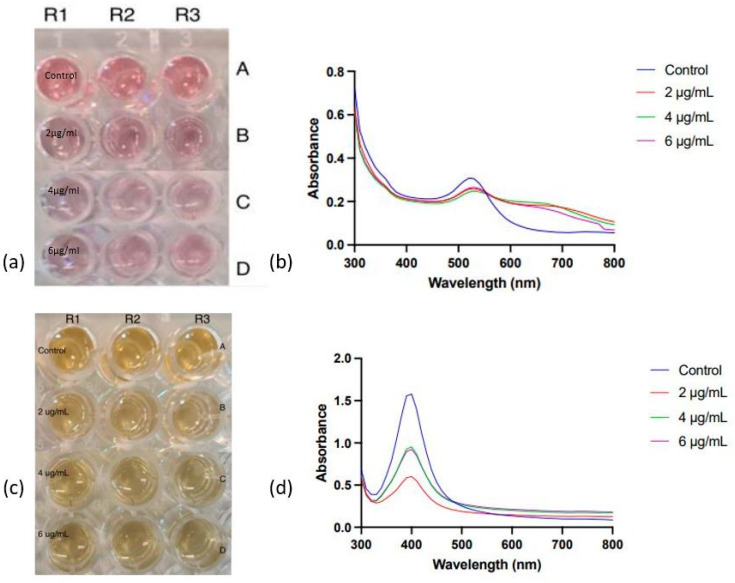
Colourimetric assay of AuNPs at varying antibody concentrations in an alkaline environment. (**a**) Row A is the control, which contains AuNPs, DI water, and antibodies. In rows B–D, the concentration of NaCl and K_2_CO_3_ was kept constant at 200/150 mM and 1.2 mM, respectively. The varying antibody concentrations were 2 and 4 µg/mL. R1, R2, and R3 are replicates. (**b**) A UV-visible spectrum of AuNPs and various concentrations of anti-zearalenone antibody conjugates. (**c**) Row A is the control, which contains AgNPs, DI water, and antibodies. In rows B–D, the concentration of NaCl and K_2_CO_3_ was kept constant at 200/150 mM and 1.2 mM, respectively. The varying antibody concentrations were 2, 4, and 6 µg/mL. R1, R2, and R3 are replicates. (**d**) A UV-visible spectrum of AgNPs and various concentrations of anti-zearalenone antibody conjugates.

This study aimed to establish the optimal antibody concentration for enhancing nanoparticle surface binding and subsequently amplifying signal output. The antibody concentration was increased in 2 µg/mL increments, and pH was controlled using 1.2 mM K_2_CO_3_ concentration. The experimentation was conducted within a 96-well plate setup, with a control comprising nanoparticles, deionised water, and varying antibody concentrations. The arrangement of nanoparticles in the plate is illustrated in Figure 6. 

AuNPs and AgNPs were conjugated with 2,4 and 6 µg/mL of anti-zearalenone antibodies at the optimised pH value of ⪆7.5. After adding the required concentration of antibodies and nanoparticles, NaCl solution was added. Nanoparticle conjugates exhibited minor colouration changes when exposed to suboptimal NaCl concentrations of 150 mM (AuNPs) and 80 mM (AgNPs). For further evaluation, OD measurements were taken at 520 nm, and variations in the OD of AuNP conjugates were evident (Figure 6b). Specifically, at 150 mM NaCl and 4 µg/mL of antibody concentration, the OD of nanoparticles (0.250) closely resembled the control sample’s OD (0.298). This suggests that the SPR was similar under these conditions, indicating successful antibody attachment. At 630 nm, a similar pattern was observed, suggesting optimal conjugation at 4 µg/mL of antibody concentration at 150 mM of NaCl. Similarly, in the case of AgNPs, the OD measurements were taken at 400 nm. However, the extent of aggregation can be regarded as acceptable, as the Ab-AuNP and Ab-AgNP conjugates retained a substantial degree of the nanoparticles’ original pinkish-red and bright yellow colour, respectively.

The influence of the varying antibody concentrations on the SPR of nanoparticle conjugates was evaluated via absorbance measurements employing UV-visible spectroscopy. In the UV-visible spectra of AuNPs-Ab conjugates (Figure 6b), the SPR peak of the control conjugates is distinct and well-defined. Conversely, the peaks corresponding to the AuNPs conjugated with 2 and 4 µg/mL of antibody concentrations are relatively broader and moved toward the higher wavelength, suggesting successful conjugation of AuNPs with the antibodies. 

In the case of AgNP-Ab conjugates (Figure 6c), the UV visible absorbance spectra display SPR peaks at 400 nm (Figure 6d). For conjugates at 2, 4, and 6 µg/mL of antibody concentrations, SPR peak intensities are lower than that of the control. Notably, 2 µg/mL exhibits the lowest intensity, while 4 µg/mL and 6 µg/mL share slightly higher intensities. An optimal antibody concentration for AgNP conjugation may not be 2 µg/mL due to the lowest SPR intensity, while 4 µg/mL and 6 µg/mL show comparable intensities, suggesting either could be optimal. Since the study’s goal is to maximise the nanoparticle-to-antibody conjugation, 6 µg/mL emerges as the optimal antibody concentration.

The OD measurements for AgNP conjugates revealed no distinct trend at 400 nm and 630 nm, likely due to comparable surface plasmon resonances among conjugates at varying antibody concentrations under controlled pH and salt conditions. Notably, control containing only nanoparticles and antibodies exhibited significantly higher OD at 400 nm compared to conjugates with varying antibody concentrations. This divergence implied differing SPR bands, possibly due to non-optimised conditions for these control conjugates.

The hydrodynamic diameter of AuNP-Ab conjugates at 2 µg/mL in 0 mM NaCl, 2 µg/mL in 150 mM NaCl, and 4 µg/mL in 150 mM NaCl were 10–90 nm, 150–450 nm, and 150–400 nm, respectively. These measurements indicated that the conjugates exhibited larger sizes compared to the control. The same pattern was observed for AgNP-Ab conjugates where the size distribution of the control and conjugates at 2 µg/mL, 4 µg/mL, and 6 µg/mL ranged from 1 to 110 nm, 1–500 nm, 150–350 nm, and 195–500 nm, respectively. These observations strongly demonstrate the effective binding of antibodies to nanoparticles within the controlled pH environment. ZP and PDI of AuNPs at varying NaCl and Ab concentrations are detailed in Table 4 and Table 5. 

ZP measurements indicated comparable surface charges between control and antibody conjugates at 4 µg/mL of antibody concentration in 150 mM of NaCl, with values of −30.9 mV and −30.53 ± 1.80 mV, respectively. These highly negative readings denote substantial stability. In contrast, nanoparticle conjugates at 2 µg/mL of antibody in 150 mM of NaCl exhibited markedly lower negative ZP value of −0.529 ± 0.54 mV. This weak ZP signifies insufficient antibody coverage on nanoparticle surfaces, causing interactions with NaCl ions and the formation of large and destabilised nanoparticle aggregates. The low ZP aligns with DLS data, revealing larger particle sizes for the sample with 2 µg/mL of antibody concentration in 150 mM of NaCl. Additionally, the highest PDI appeared at 4 µg/mL of antibody in 150 mM of NaCl, indicative of increased heterogeneity and broad size distribution in these conjugates.

These findings effectively underscore that a more efficient conjugation process can be achieved at 4 µg/mL of antibody concentration in 150 mM NaCl with 1.2 mM of K_2_CO_3_ concentration. A similar study was conducted by Kasoju et al. [12], where AuNPs stability was examined using varying NaCl concentrations ranging from 20 mM to 200 mM. The aggregation pattern observed by the researcher was identical to the pattern observed in this study, where the nanoparticles at low NaCl concentration are stable and exhibit no colour change. Contrarily, at high concentrations of NaCl, the physical stability of the nanoparticles is affected, resulting in the formation of aggregates. The ZP of AgNP-Ab conjugates at 2 and 4 µg/mL of antibody concentrations were 0.329 ± 0.16 mV and 0.403 ± 0.639 mV, respectively. These lowly positive values typically suggest the potential destabilisation of conjugates in the reaction mixture. However, this contradicts the well-defined SPR peaks and comparable OD values, both indicative of conjugate stability. The discrepancy may stem from potential contamination during reaction mixture preparation or the conjugates’ quality compromised due to prolonged room temperature exposure during analysis. The conjugates might have lost stability, possibly due to antibody sensitivity to temperature fluctuations in the environment and their requirement for low temperatures to preserve stability.

Conversely, in AgNPs at 6 µg/mL of concentration, the conjugates displayed a notably negative ZP value of −36 ± 2.11 mV, underscoring robust stability under optimised pH and salt conditions. This strongly implies enhanced temperature resistance compared to the 2 µg/mL and 4 µg/mL of antibody concentration conjugates, indicative of enhanced shelf life and sensitivity for biosensing. Hence, the optimal antibody concentration for AgNPs-Ab conjugation is 6 µg/mL. Bélteky et al. [31] performed a comparable study on AgNPs to determine the effect of NaCl on the colloidal stability of AgNP suspension. The results of the study also concluded that the SPR of the AgNPs declined with increasing NaCl concentration. The reasoning behind this is that the relative permittivity of the surrounding medium is increased with increasing NaCl concentration, thereby affecting the SPR [32]. Moreover, Bélteky et al. [31] observed changes in SPR at 10 mM and 50 mM of NaCl for AgNPs that were 10 nm and 20 to 50 nm, respectively, while in this study, this pattern was observed at 80 mM NaCl for AgNPs that are 2.5 to 120 nm. Therefore, this indicated that higher NaCl concentrations may be required to induce mild aggregation of larger nanoparticles.

Lou et al. [19] conducted a comparable investigation to determine an appropriate K_2_CO_3_ and antibody concentration for developing stable antibody-labelled AuNPs. The results demonstrated that the stability of the conjugates improved as the antibody concentration increased. This was because the nanoparticles were saturated by the antibodies, and hence the conjugates were less sensitive to NaCl and K_2_CO_3_ in the reaction mixture, preventing aggregation. A similar pattern was also observed in this study, where AuNP and AgNP conjugates were stable when the antibody concentration increased from 2 µg/mL to 4 µg/mL and 6 µg/mL, respectively. It was also found that a higher pH value is required for conjugating smaller-sized nanoparticles with antibodies compared to larger ones. This contrasted with the results of this study, as a higher pH value was required for developing stable AuNP and AgNP conjugates using Au and Ag NPs with a large hydrodynamic diameter of up to 120 nm.

## 4. Conclusions

In summary, gold and silver nanoparticles’ anti-zearalenone antibody conjugates were successfully optimised. Standard gold and silver nanoparticles were synthesised using trisodium citrate and sodium borohydride, respectively, as reducing agents. AuNPs and AgNPs exhibited SPR peaks at 520 nm and 400 nm on the UV-visible absorbance spectra, respectively, which are the characteristic peaks of the nanoparticles. SEM analysis revealed that the AuNPs and AgNPs have a spherical morphology and are estimated to be between 5.78 nm to 11.8 nm and 30 nm to 150 nm, respectively. DLS analysis further confirmed the size distribution of AuNPs and AgNPs to be 9–120 nm and 2.5–120 nm, respectively. They exhibited highly positive ZP measurements of −26.3 ± 4.6 mV and −20.07 ± 0.5 mV, confirming their high stability in the colloidal solution. Furthermore, the AuNPs and AgNPs had PDI measurements of 0.209 and 0.564, respectively, indicating that AgNPs have a comparatively broader size distribution. In the NaCl study of the AuNPs and AgNPs, the NaCl concentration at which the onset of nanoparticle aggregation occurred was determined to be 150 mm and 80 mM, respectively. This was demonstrated by changes in the AuNP and AgNP colour from red to pink-purple to blue and bright yellow to pale yellow to grey, respectively, indicating the aggregation of nanoparticles. The broadening of SPR peaks plus a decrease in peak intensities, change in ODs, increase in nanoparticle size, lowly negative ZP values, and high PDI confirmed the aggregation behaviour of the nanoparticles. In the K_2_CO_3_ study, successful Au and Ag nanoparticle–antibody conjugation was achieved at 1.2 mM K_2_CO_3_ concentration (pH ≈ 7). In the final study, it was found that 4 µg/mL and 6 µg/mL are the optimum antibody concentrations for achieving maximum Au and Ag nanoparticle–antibodies conjugation, respectively, which can help to improve the sensitivity, reproducibility, and limit detection of the lateral flow immunochromatographic assays.

## Figures and Tables

**Table 1:** ZP and PDI of standard gold and silver nanoparticles measured using DLS.

	Gold Nanoparticles	Silver Nanoparticles
ZP (mV)	−26.3 ± 4.6	−20.07 ± 0.5
PDI	0.209	0.564

**Table 2:** ZP and PDI of gold nanoparticles in controls and at 1.2 mM of K_2_CO_3_ concentration.

K_2_CO_3_ Concentration	ZP (mV)	PDI
Control 1	−33.4 ± 8.2	0.229
Control 2	−30.9 ± 10.6	0.329
1.2 mM	−23.2 ± 1.5	0.347

**Table 3:** ZP and PDI of silver nanoparticles in controls and at 1.2 mM of K_2_CO_3_ concentration.

K_2_CO_3_ Concentration	ZP (mV)	PDI
Control 1	−32.7 ± 0.26	0.179
Control 2	−31.5 ± 5.20	0.166
1.2 mM	−33.6 ± 1.11	0.312

**Table 4:** ZP and PDI of gold nanoparticles at varying NaCl and Ab concentrations.

NaCl and Ab Concentration	ZP (mV)	PDI
Control	−30.9	0.329
150 mM NaCl and 2 µg/mL Ab	−0.529 ± 0.54	0.362
150 mM NaCl and 4 µg/mL Ab	−32.5 ± 4.23	0.358
150 mM NaCl and 6 µg/mL Ab	−30.53 ± 1.80	0.411

**Table 5:** ZP and PDI of silver nanoparticles at varying NaCl and Ab concentrations.

AgNPs (NaCl + Ab)	ZP (mV)	PDI
Control	−31.5 ± 0.72	0.166
150 mM NaCl and 2 µg/mL Ab	−22.9 ± 0.16	0.283
150 mM NaCl and 4 µg/mL Ab	−32.3 ± 0.63	0.345
150 mM NaCl and 6 µg/mL Ab	−36.01 ± 2.11	0.404

## Data Availability

The data presented in this study are available upon request from the corresponding author.
